# Researches into the Nature and Cause of Epilepsy, as Connected with the Physiology of Animal Life, and Muscular Motion; with Cases Illustrative of a New and Successful Method of Treatment

**Published:** 1819-10-01

**Authors:** 


*Researches into the Nature and Cause of Epilepsy, as connected with the Physiology of Animal Life, and Muscular Motion; with Cases illustrative of a new and successful Method of Treatment.* Dy J. G. MANSFORD, &c. Author of an Inquiry into the Influence of Situation on Pulmonary Consumption, &c. One Volume Octavo, 162 pages. London, 1819.

EPILEPSY has ever been a disease so extraordinary and frightful in its appearance; so obscure in its nature; and so rebellious to treatment, that the Ancients attributed it to the anger of the Gods. Hippocrates, indeed, endeavoured to combat this idea, but still applied to it the appellation of *Morbus Sacer.* Like as in phthisis, cancer, and some other opprobria medicorum, the *successful*—nay the *infallible* methods of treating epilepsy, would fill a volume; yet, in a great majority of instances, it still defies the physician's skill, and preserves a head-strong course towards the destruction both of the mental and corporeal functions. Under such circumstances we need not wonder that every *New Remedy* should " strut and fret its hour upon the stage," even should it be used no more. It is, therefore, our duty to present our readers with an early analysis of any work which holds out confident hope in so deplorable a malady as epilepsy; while at the same time, we shall be very sparing of *opinions* on the subject, lest we should be accused of biassing the mind before a fair trial be given to the remedial measure introduced.

Mr. Mansford, in the early chapters of the work, enters into *meta*physics; a subject which he thinks essentially connected with the pathology of the disease under consideration. We shall not follow him into that airy region; for this simple reason, that we believe *first causes* to be not only *meta*-physical, but *meta*-comprehensible. They appear to us not only unprofitable, but *dangerous* studies to be indulged in, by the practical physician or surgeon, as some recent events have abundantly proved.

We may state, however, that our author considers life, "as the *union* of an immaterial principle with organized, but inert matter, of which it is independent, and over which it presides." He also believes the nervous and electric fluids to be the same, or at least that they may, for every physiological purpose, be considered as such. The voluntary movements of the body then, according to our author, are the result of a subtle and mobile matter, "transmitted by an act of the immaterial principle from the brain to the muscles," the electrical conditions of which are different. The electric or nervous fluid being imparted to the muscles, causes them to contract, and then flies back to the brain or spinal marrow, by the blood or some other medium not yet determined on. The balance between the formation and expenditure of this fluid being sometimes destroyed,

> "An accumulation disproportioned to the existing capacity of the brain, must happen, productive, perhaps, of various phenomena; but chiefly of the regular explosions, if he may so term them, of epilepsy." P. 22.

Passing over much theory; perhaps very ingenious, but we fear somewhat too *aerial,* we come to the fourth Chapter, which brings us back to the dull consideration of facts, instead of hypotheses.

Mr. Mansford does not enter into the symptomatology of epilepsy, but refers to systematic works for its description and history. He conceives that there may be two states of the brain giving rise to the disease. The *first,* where its organization is not visibly altered, but where the organ cannot bear "the excitement of the electric stimulus." The *second,* where there is an absolute change of structure, "giving rise to a morbid accumulation of the nervous fluid, or diminishing its capacity for the natural proportion." In short, our author comes to the same conclusions, though in somewhat different terms, as some late writers, that epilepsy is determined by superexcitement of the nervous, and plethora of the vascular system of the brain; which states are induced by various causes, acting either on mind or body. For illustrations of the *increased momentum* of blood [a bad expression by the bye] to the head in epilepsy, our author quotes the observations of Dr. Parry, who traces a strong affinity between this disease and head-ache, epistaxis, hysteria, hydrocephalus, &c. Mr. Mansford affirms that, in every instance, where more than one in a family were affected with epilepsy, he found some of the other branches prone to one or other of the diseases alluded to. In families of this kind, too, the epileptic affection is exceedingly difficult of cure.

Our author considering "an accumulation of electric matter in the brain, excessive with respect to its existing capacity," as the proximate cause of epilesy, was naturally led to the idea of preventing or removing this accumulation, as the basis of its hygiene and treatment; and "the galvanic power appeared to hold out the only means of accomplishing this object." For this purpose, "it was desirable that a *negative* point should be established as near the brain as possible, and a *positive* one in some distant part of the body; which should preserve their opposite states, and be kept constantly in action." The following description of Mr. Mansford's apparatus, and of the mode of its application, we shall give in the author's own words.

"A portion of cuticle, the size of a sixpence, being removed, by means of a small blister, in the back of the neck, as close to the root of the hair as possible, and a similar portion in the hollow beneath, and on the inside of the knee, as the most convenient place; to the wound in the neck a plate of silver, varying according to the age of the patient, from the size of a sixpence to that of a half crown, was applied; having affixed to its back part a handle or shank, and to its lower edge, and parallel with the shank, a small staple, to which the conducting wire was fastened. This wire descended the back, till it reached a belt of chamois leather, buttoned round the waist; it then followed the course of the belt, to which it was attached, till it arrived opposite the groin, on the side it was wished to be used; it then passed down the inside of the thigh, and was fastened to the zinc plate in the same manner as to the silver one. The apparatus so contrived, was thus applied:—a small bit of sponge moistened in water, and corresponding in size to the aperture in the neck, was first placed directly upon it; over this a larger piece of sponge of the same size as the metallic plate, also wetted, was laid; and next to this the plate itself, which was secured in its situation by a strip of sticking plaster passed through the shank on its back, another above, and another below it. If these be properly placed, and the wire which passes down the back, be allowed sufficient room that it may not drag, the plate will not be moved from its position by any ordinary motion of the body. The zinc plate was fastened in the same manner; but in place of the second layer of sponge, a bit of muscle, answering in size to the zinc plate, was interposed; that is, a small bit of moistened sponge being first fitted to the aperture below the knee, the piece of muscle also wetted then followed, and on this the plate of zinc. The apparatus thus arranged will continue in gentle and uninterrupted action from twelve to twenty-four hours, according to circumstances. This last is the longest period that it can be allowed to go unremoved:—the sores require cleaning and dressing, and the surface of the zinc becomes covered with a thick oxyde, which must be removed to restore its freedom of action; this may be done by scraping or polishing; but it will be better if removed twice a-day, both for the greater security of a permanent action, and for the additional comfort of the patient." P. 88.

The above is Mr. Mansford's apparatus for conducting electricity from the higher to the lower regions of the body, and securing the *capital* from the explosions of this all-powerful element. We shall make a few observations on it presently.

Meantime our author informs us, that the plan above mentioned does not preclude the use of other appropriate remedies. All *exciting causes* must, if possible, be removed; and particularly vascular fulness, by means of small bleedings, repeated at intervals, according to the tendency to a return to excessive action. Our author has "been fond, in such cases, [topical plethora] of drawing blood by means of cupping glasses from the parts immediately over the cervical and superior dorsal vertebræ." He also recommends dry-cupping, as a valuable auxiliary. Next to the state of the circulation, the state of the digestive organs is to be attended to; and as there is generally torpor of the alimentary canal, their secretions and peristaltic motion ought to be invited by small doses of aloetic compositions, or other eccoprotics, so that one or two evacuations should be procured daily, while temperance is to be strictly enjoined. Here Mr. Mansford makes some judicious observations on the *juvantia et lædentia* in epilepsy, but which are known to most of the well informed classes of medical society. The detail of nine cases of the disease follows. We shall give a condensed statement of a few of these.

*Case* 1. Louisa Trotman, ætat. 11, was subject to epileptic fits, since the age of six years. They almost always occurred about three o'clock in the morning, and lasted with strong and uninterrupted convulsions till eight. For some days after each attack, the little patient was in a state of perfect mania, talking or singing incessantly, with great irascibility. This state was succeeded by stupor and somnolency. The usual interval between the fits was from three to five weeks; if longer, the paroxysms were proportionally violent. The patient was, in fact, in a state of idiotcy, with evident malconformation of the head. Various remedies had been tried in vain. On the 29th of May, 1816, our author applied the galvanic apparatus, above described, at first only during the night, but, after the first fortnight, constantly.

"It may be imagined to have been a source of the highest gratification to the parents and to myself, to observe the period of the fit pass by, without even any of the symptoms which usually indicated its approach."

About the middle of the tenth week of this happy suspension of epilepsy, our author was summoned to the patient in a fit. The mother of the child, however, soothed the mortification of our author, by informing him, that "he need not be discouraged, for that the connecting wire had broken in the night."

"I immediately, (says Mr. M) thought of trying the effect of an increased galvanic power. Accordingly a small pile, consisting of twelve pairs of plates with intervening bits of cloth moistened with a solution of the muriate of ammonia, was included in the animal circle; its negative end communicating with the wound in the neck, and the positive one with that in the leg. About ten minutes after this was put in action the fit ceased, and gave place as usual, to a sound sleep." P. 112.

The mother of the child dying soon after this, the apparatus was neglected, and a prospect of cure was abandoned.

*Case* 2. John Rice, ætat. 12, in the employ of Mr. Barret, of Bath, had suffered, for several months, from irregular attacks of epilepsy, increasing in frequency, till they became daily occurrences. Several respectable practitioners in Bath were foiled in their attempts to arrest the disease. The boy was sent to our author at Frome. The galvanic process, as before described, completely effected his cure in little more than two months.

The other seven cases detailed, tend to the same conclusions as the above, and, therefore, we think it unnecessary to present any analysis of them here.

When we recollect that the *cause* of the epileptic paroxysm has its *domicile,* if we are allowed the expression, occasionally, in every organ or tissue of the body, we can entertain but weak hopes of ever finding out a *specific* remedy for the disease. The great art is to ascertain this *domicile;* for it is by attacking it *there* that we have any chance of success. In respect to the remedy here brought forward by Mr. Mansford, we confess our scepticism of its *physical* power over the malady. We know how much epilepsy is under the influence of *moral* emotions; and as the apparatus delineated is calculated to make a considerable impression on the mind of the patient, so, in conjunction with the constitutional treatment, we have no doubt that the effects of its application were such as Mr. Mansford has stated. The *counter-irritation* too may have had a share in the fortunate results. We much fear that *in this way only* can the suspension of epilepsy be accounted for, in Mr. Mansford's practice, and that this suspension will prove but temporary.

We are ready to grant, however, that Mr. Mansford has evinced considerable ingenuity, both in the theoretical and practical parts of the volume; and that though the anti-epileptic apparatus wears much the semblance of an amulet, it may, in the author's hands at least, be productive of some benefit.

